# Effect of Thermal Simulation Process on Microstructure of Seismic Steel Bars

**DOI:** 10.3390/ma15103438

**Published:** 2022-05-10

**Authors:** Sheng Huang, Changrong Li, Zhiying Li, Changling Zhuang, Zeyun Zeng, Jie Wang

**Affiliations:** 1 College of Materials and Metallurgy, Guizhou University, Guiyang 550025, China; hs19967691957@163.com (S.H.); lizhiying2015@163.com (Z.L.); clzhuang@gzu.edu.cn (C.Z.); zzy19950406@163.com (Z.Z.); 2 Guizhou Province Key Laboratory of Metallurgical and Process Energy Saving, Guiyang 550025, China; 3 Rolling Business Department, Shougang Shuicheng Steel, Liupanshui 553000, China; clyyjxy520@126.com

**Keywords:** micro structure, thermal simulation, high-density dislocation, precipitate

## Abstract

Thermal deformation has a significant influence on the microstructure of high-strength antiseismic steel. The effect of hot deformation on the microstructure of experimental steel was studied by the Gleeble-3800 thermal simulator. The microstructure of the steel was characterized by the metallographic microscope, microhardness, tensile test, field emission scanning electron microscope, electron backscatter diffraction, and high-resolution transmission electron microscope. The results show that the core microstructure of the test steel is composed of polygonal ferrite and lamellar pearlite. The test steel is mainly ductile fracture. Tensile strength and hardness increase with the decrease of temperature. At 650 °C isothermal temperature, the ferrite distribution was uniform, the average grain size was 7.78 μm, the grain size grade reached 11, the pearlite lamellar spacing was 0.208 μm, and the tensile fracture was distributed with uniform equiaxed dimples. Polygonal ferrite grain boundaries have high density dislocations that can effectively block the initiation and propagation of cracks. However, there are some low dislocation boundaries and subgrain boundaries in ferrite grains. Precipitation strengthening is mainly provided by fine precipitates of V-rich carbonitride in experimental steel. The precipitates are round or narrow strips, about 70–100 nm in size, distributed along ferrite grain boundaries and matrix.

## 1. Introduction

In recent decades, microalloyed high-strength antiseismic steel has been developed for the construction industry, particularly for the house and bridge construction industries [1,2]. The nature and content of microalloying elements and hot deformation have significant influences on the microstructure of microalloyed steel. Excellent and ideal mechanical properties can be obtained by optimizing alloy composition design and thermal mechanical control processes, including grain strengthening, microstructure control, and precipitation strengthening [3,4,5]. The key factors are the formation of fine austenite grains during hot deformation and transformation of undercooled austenite into fine ferrite grains during final cooling [6,7]. In the hot deformation process, grain refinement occurs in the transformation process when deformation increases [8,9]. Rolling ratio, rolling temperature, cooling method, final cooling temperature, and other process parameters have considerable impacts on the microstructure of microalloyed hot rolled steel, and final cooling temperature has the most significant impact [10].

The effect of thermal deformation on the microstructure of microalloyed (such as Nb, V, and Ti) high strength steel has been explored in many studies [11,12,13]. Several scholars found that steel with nanocarbide particles precipitated in ferrite matrix had high strength (≈780 MPa) and showed good elongation (≈23%). Complex nanocarbides have excellent thermal stability and can maintain their small sizes during final cooling. Many scholars [14,15] showed the effect of Nb on the microstructure evolution of austenite during hot deformation. Whether Nb exists in the form of solid solution or Nb (C, N) strain induced precipitation, it can delay the recrystallization softening process of austenite and lead to the refinement of austenite grains. Increase in deformation temperature inhibits Nb precipitation in austenite and promotes Nb precipitation in ferrite [16]. Shanmugam et al. [17] studied the morphology, sizes, and chemical compositions of particles formed during continuous cooling in Nb–V–Ti microalloyed steel. They divided 20–45 nm spherical precipitates into (Ti, Nb, V) C and (Nb, V) C and categorized 45–70 nm cubic particles into (Ti, Nb) N. Akhlaghi et al. [18] studied 5 nm fine NbC precipitates formed during continuous cooling in Nb–V–Ti microalloyed steel. Some scholars [19] showed that the ferrite grain, pearlite cluster size, and pearlite interlayer spacing of V–Ti–N microalloyed medium carbon steel can be refined. (V, Ti) (C, N) or (V, Nb, Ti) (C, N) precipitates are present in V–Ti or V–Ti–Nb microalloyed steel [20], and nanosized microalloyed carbonitride forms during long time holding at 450–650 °C. Carbide can markedly improve yield strength by preventing dislocation movement. Strain-induced precipitation of 20–30 nm is the nucleation center of acicular ferrite [21].

Seismic reinforcement can absorb the energy generated by the earthquake, resist the fracture disaster caused by the earthquake, and protect people’s property safety. Seismic steel bars act on high-rise buildings, bridge tunnels, and sea-crossing bridges. At present, some enterprises are faced with various factors, such as low production efficiency, unqualified technical conditions, and low profits in producing seismic reinforcement. Through microalloying and controlled rolling and controlled cooling, the performance of the seismic steel bar is optimized, and the production cost is reduced. In many studies, it was found that adding microalloying elements and controlling the final cooling temperature were more economical and effective ways to solve the problem of insufficient steel performance and increase the production efficiency of enterprises. Therefore, the thermal simulation process was explored to determine the best final cooling temperature to obtain the best microstructure and mechanical properties. It provides a theoretical basis for the development of seismic steel bars with high strength and good toughness.

## 2. Experimental Procedures

### 2.1. Preparation of Test Sample

According to China’s 2018 release of “Steel for Reinforced Concrete Part 2: Hot Rolled Ribbed Steel Bar” (abbreviation: GBT 1499.2-2018): hot rolled ribbed steel bar standard and internal control standard for high-strength antiseismic steel in a steel plant, the control range of Nb + *V* is 0.085–0.115%. The equivalent carbon content (*C_eq_*) and welding crack sensitivity index (*P_cm_*) calculated by Equations (1) and (2) are 0.462 and 0.255, respectively.

(1) $$C_{eq}=C+\frac{Mn+Si}{6}+\frac{V}{5}$$

(2) $$P_{cm}=C+\frac{Si}{30}+\frac{Mn}{20}+\frac{V}{10}$$

The raw material of the experimental steel was microalloyed high-strength antiseismic steel, which was provided by a steel plant. The composition design of microalloying elements was mainly considered, and two factors, namely conventional and microalloying elements (Nb, V, and Ti), were considered in the composition design. An ingot (15 kg) was melted in a medium-frequency induction furnace and forged into a Φ40 mm × 60 mm steel bar. Inductively coupled plasma spectroscopy/mass spectrometry (ICP-AES/MS), inorganic carbon and sulfur analyzer, inorganic oxygen, nitrogen, and hydrogen analyzer were used to test the composition of the test steel, taking into account the segregation of the sample composition. Each sample was tested with three sample components to get the average value. The chemical composition of experimental steel is illustrated in Table 1.

### 2.2. Thermal Simulation Process

The test steel wire was cut and processed into a rectangular sample of 10 mm × 15 mm × 20 mm, and a plane compression test was carried out on a Gleeble-3800 thermal simulator to simulate the hot rolling process of the enterprise.

**Figure 1:**
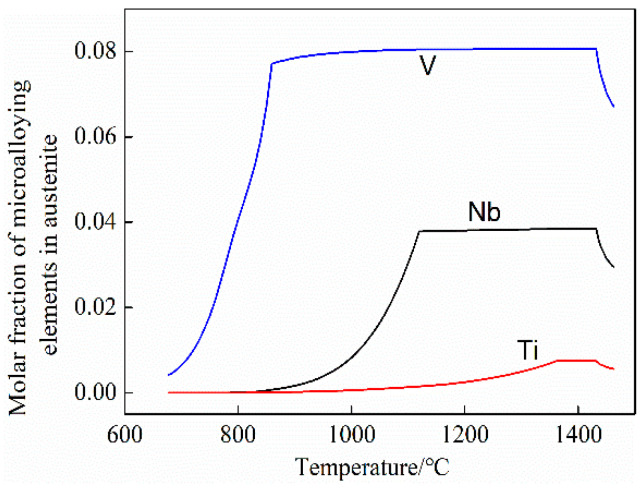
Calculated solution temperature of microalloying elements in austenite of test steel.

To find a suitable austenitizing temperature, the JMatPro software was used to calculate the solid solution of microalloying elements in austenite. The calculation results are shown in Figure 1. Therefore, the austenitization temperature in the thermal simulation process was set to 1150 °C, followed by holding for 5 min to maximize the solid solution of microalloying elements in austenite. Then, the temperature was lowered to the corresponding rolling temperature at a speed of 5 °C/s, and five passes of thermal simulation rolling were performed. The first pass temperature was 1050 °C, the deformation amount was 0.3, and the deformation rate was 2/s. Deformation temperatures during the remaining four passes were 1000 °C, 980 °C, 950 °C, and 900 °C. The deformation amount (0.1) and deformation rate (0.4/s) remain unchanged. Cooling was performed at 15–20 °C/s for the control of the final cooling temperature (650 °C, 700 °C, and 750 °C) for 30 s. Then, the morphology of ferrite and pearlite was observed. Finally, slow cooling to 200 °C at 0.5 °C/s was performed, followed by air cooling.

### 2.3. Experimental Methods

Then followed rough grinding, fine grinding, and polishing etching (4% nitric acid solution) preparation. The composition, distribution of microstructure, and pearlite lamellar morphology were observed using an OLYMPUSGX71 metallographic microscope and SUPRA40 scanning electron microscope. The viewing plane was parallel to the deformation direction. The proportion of ferrite and pearlite in the microstructure of the sample was determined using the Ipwin32 software image analyzer. The average grain size of ferrite and the lamellar spacing of pearlite were measured with a Nano Measurer image analyzer.

The samples used for EBSD and high-resolution TEM were obtained from the centers of the thermal simulation samples. EBSD samples were cut into 5 mm × 5 mm × 5 mm rectangular samples with a wire cutter. The samples were polished, then mechanically polished and electropolished (perchloric acid and ethanol solution). The scanning step size was 0.65 μm, and the scanning area was 385 × 385 μm^2^. The horizontal direction was the scanning surface. To distinguish between high-angle grain boundaries (HAGB) and low-angle grain boundaries (LAGB), grain boundaries with misorientation greater than 15° were regarded as HAGB, and misorientations less than 15° were regarded as LAGB.

The precipitation state of Nb/V/Ti, microstructure morphology, and high-density dislocation were observed in TEM samples prepared according to the standard. The thermal simulation samples were processed into Φ5 mm × 4 mm small circular plates, which were ground to 0.1 mm thickness. Mechanical thinning was performed on each sample until the thickness was 30 μm, then ion thinning was performed. The microstructures of the samples, as well as the precipitation states of the precipitates and high-resolution phases of dislocations, were analyzed with a Tecnai G2F20 S-TWIN high-resolution transmission electron microscope in bright and dark fields. The working voltage was 200 kV. The compositions of the Nb/V/Ti precipitates were analyzed by EDS equipped with TEM. The microhardness was measured on the HVS-1000 hardness testing machine, the selected loading load was 0.1 kg, the loading time was 15 s, and each sample was measured 20 times to obtain the average value. The room temperature tensile properties were tested using an electronic universal tensile testing machine, INSTRON8802, with a test speed of 1 mm/min. To ensure the accuracy of the experiment, each sample was repeated.

## 3. Results and Analysis

### 3.1. True Stress–True Strain Curve

**Figure 2:**
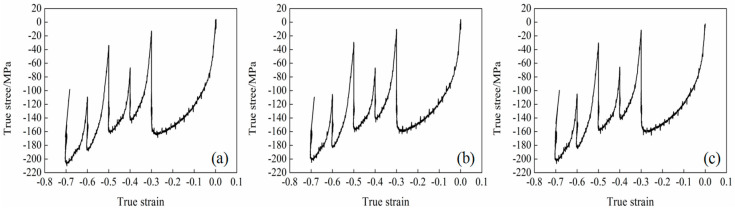
True stress−true strain curves of experimental steel at different final cooling temperatures after five passes of thermal simulation (**a**) 650 °C; (**b**) 700 °C; (**c**) 750 °C.

The true stress–true strain curve of the experimental steel was obtained by austenitization at 1150 °C for 5 min on the Gleeble-3800 thermal simulator, then the steel was subjected to plane compression for five passes, as shown in Figure 2.

It can be seen from Figure 2 that under the condition that the total rolling deformation is 0.7, the changing trend of the true stress-true strain curve of the test steel after five passes is the same. In the austenite recrystallization hot rolling zone after one pass and two passes of thermal simulation, austenite underwent dynamic recrystallization, which changes austenite grains and increases grain boundaries. In the austenite non-recrystallization hot rolling zone of three, four, and five passes, austenite shows no recrystallization and presents a long handle shape. The carbides of the microalloying elements, Nb, V, and Ti, precipitate at the austenite grain boundary. The third pass was carried out in the non-recrystallized region. With the increase of the deformation, the grains slip, and the dislocation increases, which leads to work hardening, and the true stress of the test steel gradually increases. The true stress–true strain curve usually shows the progressive work hardening of the peak stress. When the peak stress was exceeded, real stress is nearly unchanged. The absence of flow softening beyond the peak stress indicates that the steel does not undergo dynamic recrystallization and/or ferrite transformation [22].

### 3.2. Microstructure Analysis

**Figure 3:**
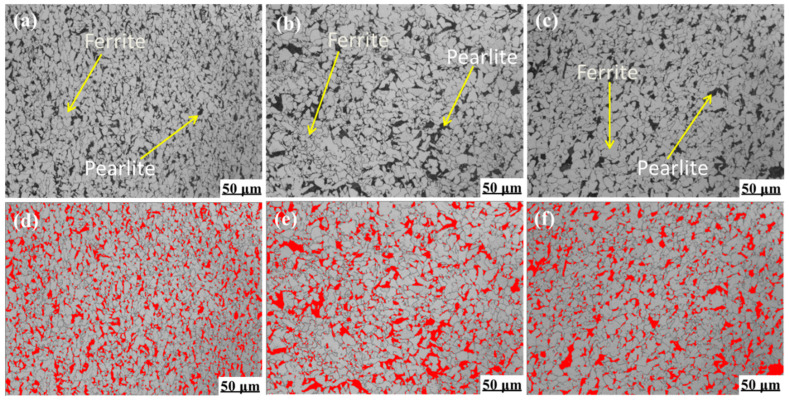
Metallographic microstructure of experimental steel under different final cooling temperatures after five passes of thermal simulation. (**a**,**d**) 650 °C; (**b**,**e**) 700 °C; (**c**,**f**) 750 °C.

The metallographic microstructure of the experimental steel under different final cooling temperatures (650 °C, 700 °C, and 750 °C) after five passes of plane compression is shown in Figure 3.

Through thermal simulation experiments, the optical microstructure of the steel is shown in Figure 3. The tested steel is mainly composed of polygonal ferrite and pearlite.

**Figure 4:**
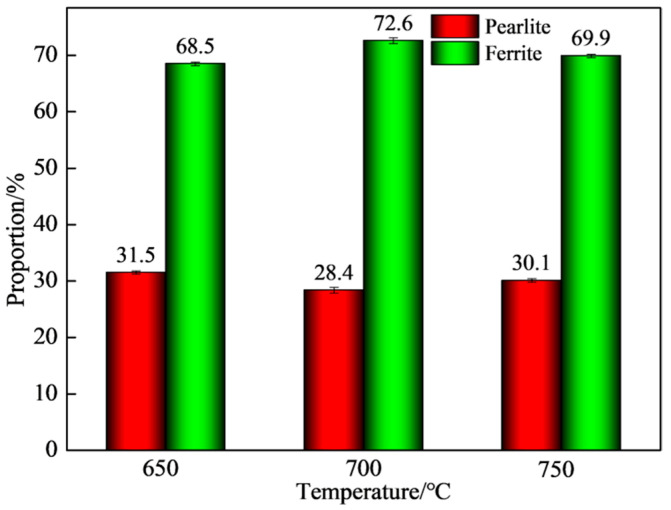
Proportion of ferrite and pearlite in the experimental steel.

The pearlite structure was processed by Ipwin32 software, and the calculation results are shown in Figure 3d–f. By calculating the same area, Ipwin32 software captured 578 ± 13, 385 ± 15, and 360 ± 10 pearlite particles at 650 °C, 700 °C, and 750 °C, respectively. Then, the area of pearlite (red area) was counted, and the area proportions of pearlite at 650 °C, 700 °C, and 750 °C were 31.5 ± 0.3%, 28.4 ± 0.5%, and 30.1 ± 0.3%, respectively. Figure 4 shows the ratio of ferrite and pearlite in the test steel at different final cooling temperatures.

The proportion of pearlite among the three final cooling temperatures was similar, and the number of particles at 650 °C was higher. Therefore, it can be seen from the viewing angle image and specific data that the pearlite distribution was relatively uniform and the grain boundaries were regular when the final cooling temperature was 650 °C. When the final cooling temperature was 700 °C and 750 °C, the ferrite grain boundary in the test steel was clear, the pearlite had a slight aggregation phenomenon, and the distribution was uneven.

**Figure 5:**
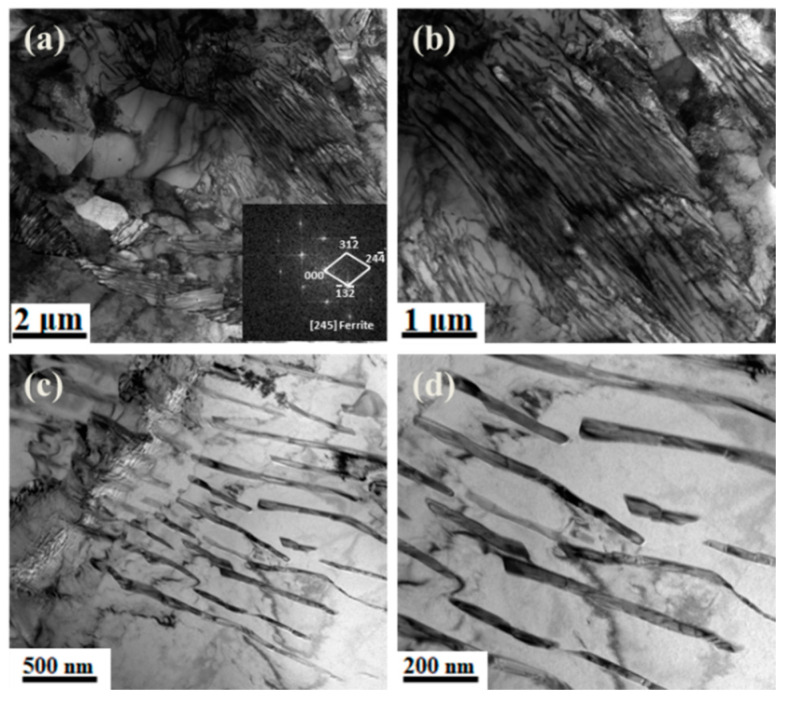
TEM morphology of ferrite and pearlite in experimental steel at final cooling temperature of 650 °C. (**a**) Morphology of ferrite and pearlite, (**b**–**d**) enlarged figure.

Figure 5 shows the TEM morphology of ferrite and pearlite of the experimental steel at a final cooling temperature of 650 °C: Figure 5a morphology of ferrite and pearlite, Figure 5b–d enlarged figure. Figure 5 shows the dislocation distribution on the ferrite and pearlite matrix of the experimental steel at 650 °C.

**Figure 6:**
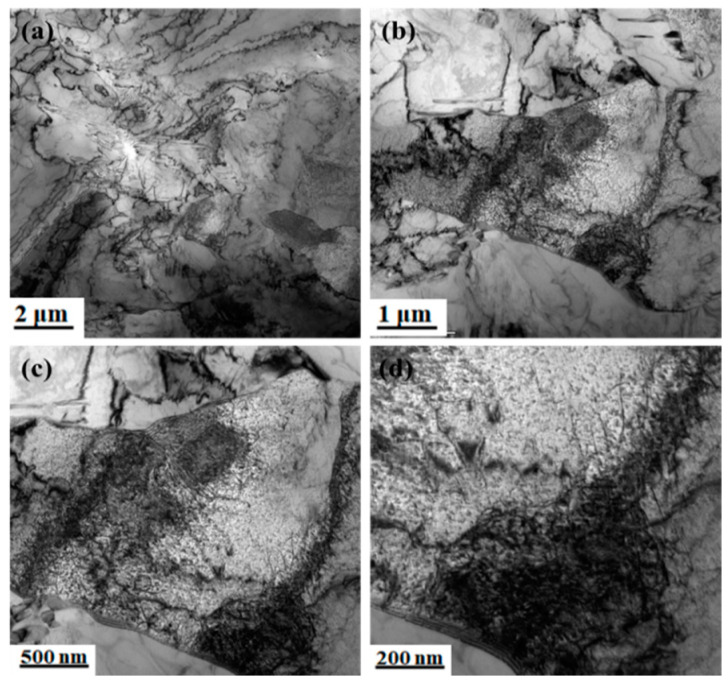
Dislocation density distribution on the matrix (650 °C). (**a**) Morphology of the dislocation density distribution, (**b**–**d**) enlarged figure.

It can be seen from Figure 5 that the ferrite of the body-centered cubic structure was polygonal, the pearlite was distributed on the ferrite matrix, and the pearlite lamellae were relatively clear and arranged in an orderly manner. Figure 6 shows that a large number of dislocations were distributed on the ferrite matrix and grain boundaries of the experimental steel, and a large number of dislocations appeared between the pearlite lamellae. Owing to the recrystallization and non-recrystallization of deformed austenite and the effect of microalloying elements (Nb/V/Ti) after five passes of hot rolling, the grain boundaries increased and a large number of dislocations were produced.

Table 2 show the statistics of the grain size and grain size grade of the test iron ferrite. The grain size of the steel at different final cooling temperatures was calculated by the intercept method (according to ASTM). The ferrite grain sizes of the experimental steels were 7.68 ± 1.3 μm, 10.67 ± 1.5 μm, and 17.07 ± 1.6 μm, and the grain sizes were 11 ± 0.25, 10 ± 0.25, and 9 ± 0.25, respectively (The results are shown in Table 2). Therefore, the smallest ferrite grain size of the experimental steel and highest grain size grade were obtained at 650 °C final cooling temperature. The results show that microalloying elements Nb/V/Ti precipitate after five passes of hot rolling, which can inhibit the recrystallization of deformed austenite and refine ferrite grains.

**Figure 7:**
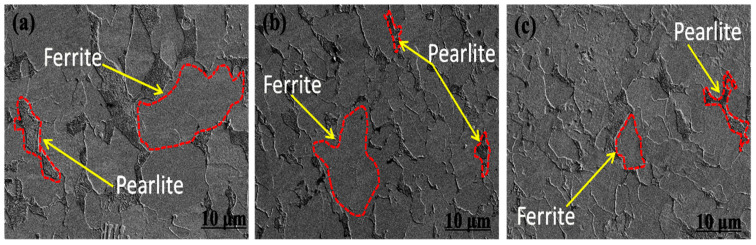
SEM microstructure of experimental steel with different final cooling temperature. (**a**) 650 °C; (**b**) 700 °C; (**c**) 750 °C.

Figure 7 shows the SEM microstructure of the experimental steel under different final cooling temperatures (650 °C, 700 °C, and 750 °C) after five passes of thermal simulation.

Figure 7 shows that the microstructure of the experimental steel was composed of polygonal ferrite and lamellar pearlite structures, and pearlite was distributed on the ferrite grain boundary regardless of the final cooling temperature. In the process of austenite hot rolling, microalloying elements Nb and Ti precipitate at the austenite grain boundary, inhibit austenite grain growth, and refines austenite grain. In the process of γ→α transformation, fine ferrite grains were obtained, and a large amount of V precipitated at the ferrite grain boundary, which acted as a heterogeneous nucleation core of polygonal ferrite and, thus, refined ferrite grains.

**Figure 8:**
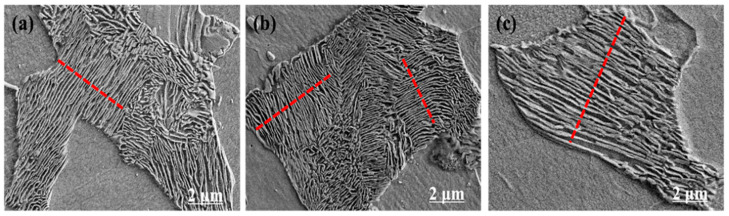
Pearlite lamellar morphology of the experimental steel at different final cooling temperatures after five passes. (**a**) 650 °C; (**b**) 700 °C; (**c**) 750 °C.

Figure 8 shows the morphology of the pearlite lamellar spacing of the experimental steel under different final cooling temperatures (650 °C, 700 °C, and 750 °C) after five passes of thermal simulation.

Figure 8 shows that the morphology of the pearlite lamellar spacing in the experimental steel at different final cooling temperatures are roughly the same, and lamellar spacing is relatively thin. When the final cooling temperature was 650 °C, the lamellar spacing of pearlite was relatively thin and evenly distributed on the ferrite grain boundary. When the final cooling temperature was 750 °C, the pearlite lamellar spacing was large, and some lamellar spacing was interrupted. During five passes of hot rolling, austenite deforms in recrystallized and non-recrystallized regions. Many defects appeared on austenite grain boundary, there was high energy, and atoms easily diffused. These conditions were conducive to fluctuations in composition, energy, and structure because of the slow cooling rate in the core of the experimental steel. Most of the cores of cementite or ferrite form on these austenite crystal defects, and interlaced cementite and ferrite form lamellar pearlite. In addition, the precipitation of the carbides and nitrides of microalloying elements Nb/V/Ti inhibits the growth of austenite grains and refines the ferrite grains and pearlite lamellae during γ → α transformation.

According to the statistics of Nano Measurer software image analyzer, the lamellar spacing values obtained through pearlite refinement in the experimental steel at 650 °C, 700 °C, and 750 °C final cooling temperatures were 0.208, 0.214, and 0.236 μm, respectively. When the final cooling temperature was 650 °C, the pearlite lamellar spacing was the smallest in the experimental steel because of deformation of austenite during five passes of hot rolling, the precipitates of Nb/V/Ti segregating, and precipitates on the grain boundaries, dislocations, and defects. The austenite grain boundaries were pinned, and thus the growth of austenite grains was hindered, and the pearlite lamellae was refined in the process of slow cooling γ → α transformation.

**Figure 9:**
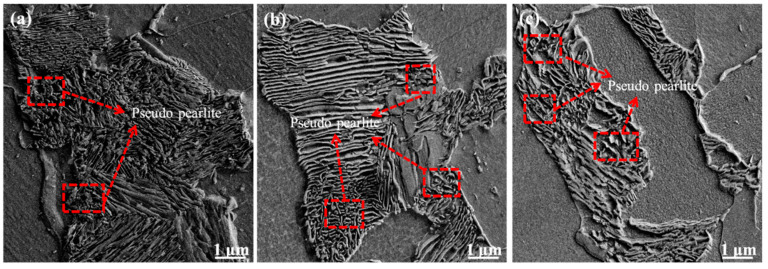
Morphology of pseudo pearlite of experimental steel. (**a**) 650 °C; (**b**) 700 °C; (**c**) 750 °C.

Pseudo pearlite is formed by pearlite from undercooled austenite deviating from eutectoid composition. Figure 9 shows that the morphology of the pseudo pearlite formed in the experimental steel at different final cooling temperatures and after five passes of thermal simulation.

Figure 9 shows that the morphology of pseudo pearlite formed in the experimental steel at different final cooling temperatures (650 °C, 700 °C, and 750 °C) is the same, has an irregular short rod or granular shape, and is disorderly distributed on the ferrite matrix. The reason is that the cooling rate of the center and edge of the steel varied under different final cooling temperatures, resulting in temperature difference, increase in the degree of undercooling, uneven distribution of carbon atoms in undercooled austenite, and slow diffusion rate. Moreover, transformed ferrite carbon atoms do not easily diffuse and migrate to long distances, pearlite lamellae gradually decrease in number and are far away from eutectoid composition, and pearlite transformation increasingly deviates from the equilibrium eutectoid point. When the transformation temperature was lower than the eutectoid point temperature, cementite cannot easily form continuous flakes. Most cementitious are distributed on the ferrite matrix and have irregular granular or short-rod shapes, forming pseudo pearlite. Relevant studies [23,24,25,26] showed that the formation process of pseudo pearlite in low carbon steel is the degradation process of pearlite, and both occur simultaneously and promote each other.

### 3.3. EBSD Analysis

**Figure 10:**
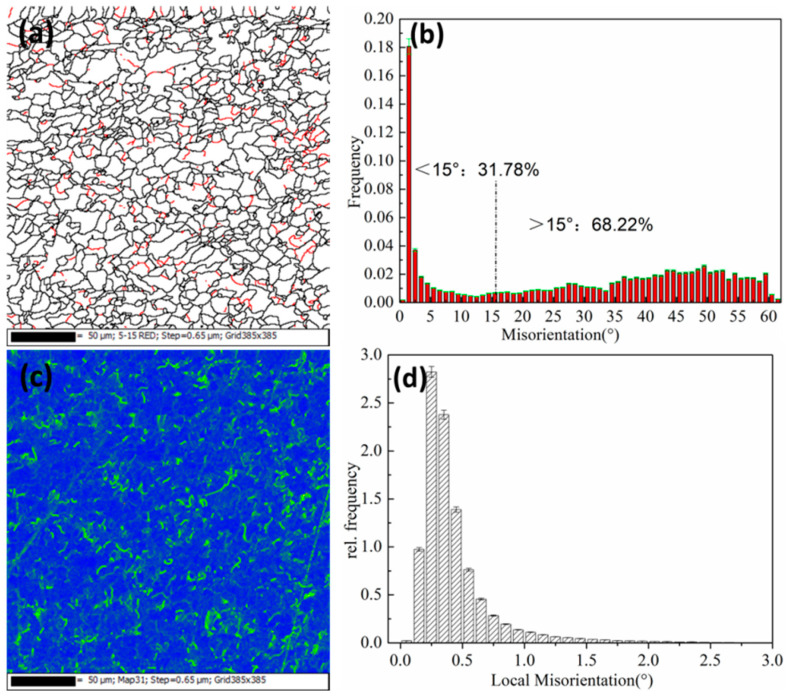
Grain boundary map and local orientation difference at 650 °C. (**a**,**b**) Grain boundary maps; (**c**,**d**) Local orientation difference.

**Figure 11:**
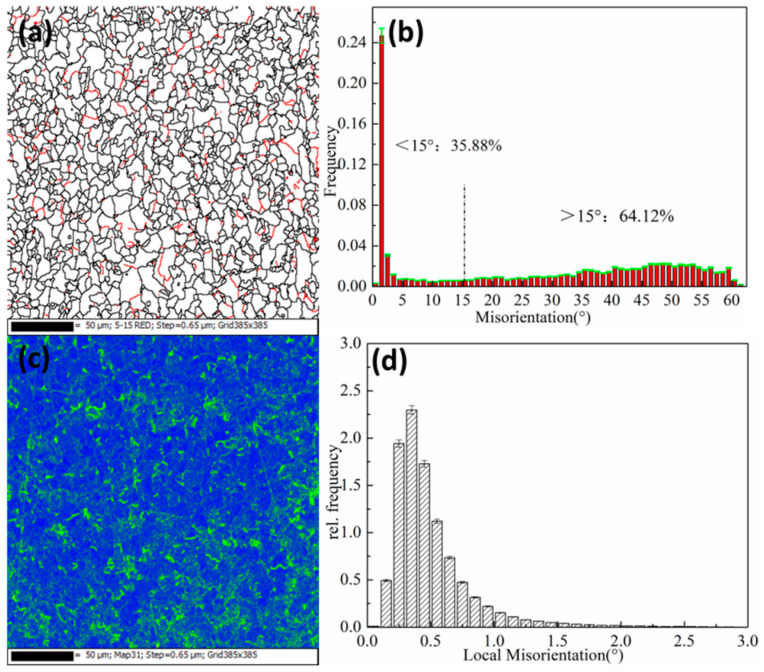
Grain boundary map and local orientation difference at 750 °C. (**a**,**b**) Grain boundary maps; (**c**,**d**) Local orientation difference.

The electron backscatter diffraction (EBSD) scanning technique were used to characterize the microstructure of the test steel. As shown in Figure 10 and Figure 11, the size and angle grain boundary distribution map and the local misorientation distribution of the experimental steel at 650 °C and 750 °C were obtained. When greater than 15°, it was regarded as a large-angle grain boundary (indicated by the black line); and less than 15°, it was regarded as a small-angle grain boundary (indicated by the red line).

The high-angle grain boundary can effectively inhibit crack propagation and improve the toughness of the material. It can be seen from Figure 10a,b and Figure 11a,b that the large-angle at 650 °C has a higher proportion than that at 750 °C, and the toughness is better. Low-angle grain boundaries are formed during recrystallization and pressure working. Small-angle grain boundaries are mainly distributed in polygonal ferrite crystals and near subgrain boundaries, accounting for 31.78% at 650 °C and 35.88% at 750 °C. Small angles are representative of the dislocation structure, and during the deformation of austenite during roughing and finishing rolling, the phase transformation leads to an increase in the dislocation structure density. Figure 10c,d and Figure 11c,d show the local misorientation distribution results after five passes of rolling. The defect and dislocation density of the samples can be seen. A green color appears around the grain boundaries, with concentrated defects and strains. The deformation first occurs during rolling deformation, resulting in a large number of dislocations around the grain boundaries. The error density was relatively large, which is not conducive to plastic deformation.

### 3.4. Mechanical Property

**Figure 12:**
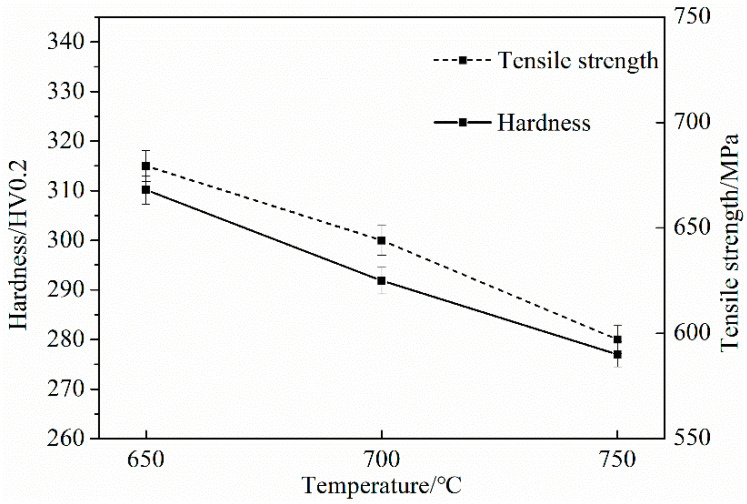
Tensile strength and microhardness of test steel.

Figure 12 shows the tensile strength and microhardness curves of the samples. It can be seen from Figure 12 that the tensile strength was proportional to the microhardness.

**Figure 13:**
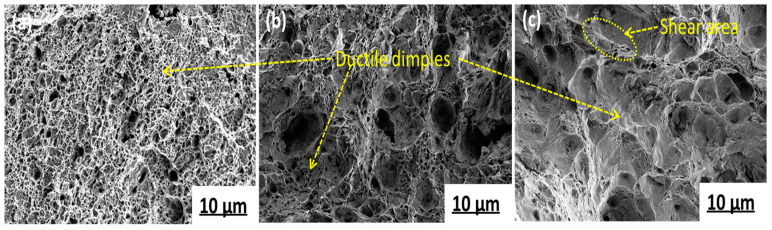
Tensile fracture morphology of test steel. (**a**) 650 °C; (**b**) 700 °C; (**c**) 750 °C.

As the temperature decreased, the tensile strength and hardness decreased. When the sample was subjected to external force, the elastic strain accumulated continuously. When the sample overcame the bonding force between the second phase particles and the matrix, a new surface was formed. Finally, a dimple-like fracture morphology was formed. The tensile fracture microstructures of the three samples at different temperatures are shown in Figure 13.

It can be seen from Figure 13 that at 650 °C, the fracture presents aggregated dimples and is an evenly distributed equiaxed dimple. At 700 °C, the size of the dimples varied, and some individual dimples were large and non-uniform. At 750 °C, there were small dimples and some small shear planes on the fracture surface. In summary, when the final cooling temperature was 650 °C, the microstructure distribution was uniform, and the large-angle grain boundaries account for a large proportion, which is good for the plasticity of the material. According to the Hall–Petch formula, the grain refinement made the hardness and strength high, the pearlite lamellar spacing also had the effect of improving the strength, and the nanometer size in the matrix played a certain role in the tensile strength of the steel. The precipitation strengthening effect of the second phase in the steel at 650 °C is analyzed below.

### 3.5. Characterization of Precipitates

**Figure 14:**
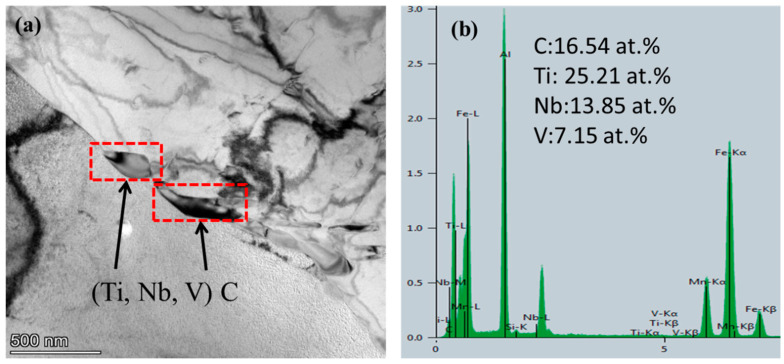
Images of (Ti, Nb, V) C precipitates in the experimental steel at 650 °C after five passes of thermal simulation. Images of (**a**) (Ti, Nb, V) C precipitates and (**b**) EDS of Nb, V, and Ti.

**Figure 15:**
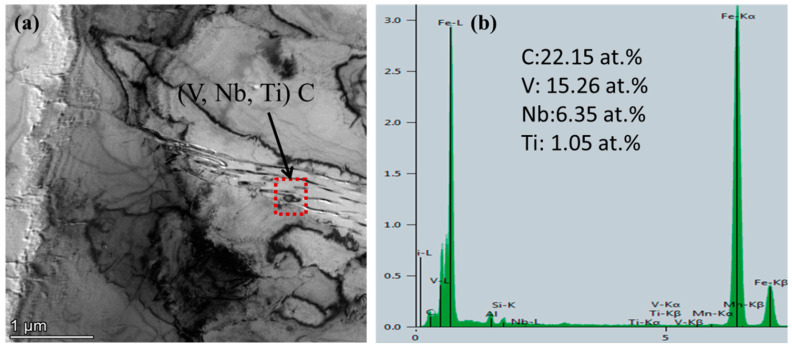
Images of (V, Nb, Ti) C precipitates in the experimental steel at 650 °C for five passes of thermal simulation. Images of (**a**) (V, Nb, Ti) C precipitates and (**b**) EDS of Nb, V, and Ti.

**Figure 16:**
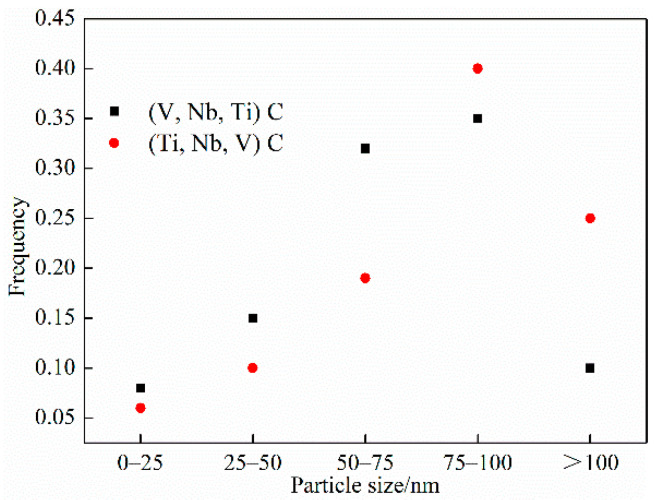
Proportion of precipitates in the experimental steel at a final cooling temperature of 650 °C.

For the analysis of the precipitation state of Nb/V/Ti in experimental steel. Figure 14 shows that the images of (Ti, Nb, V) C precipitates in the experimental steel when the final cooling temperature was 650 °C after five passes of thermal simulation. Figure 15 shows that the images of (V, Nb, Ti) C precipitate in the experimental steel after five passes of thermal simulation and at a final cooling temperature of 650 °C. Figure 16 shows the proportion of precipitates in the experimental steel when the final cooling temperature is 650 °C.

Figure 14 shows that when the final cooling temperature is 650 °C and the final cooling temperature is 650 °C, (Ti, Nb, V) C precipitates at the ferrite grain boundary, which is a long strip with a width of about 70–100 nm. It can effectively pin the austenite grain boundary and prevent the austenite from coarsening and moving. Figure 14b shows that the Ti-rich precipitates at the ferrite grain boundaries are mainly Nb/V/Ti, and the atomic ratio of Ti:Nb:V is 25.21:13.85:7.15.

Figure 15 shows that when the final cooling temperature is 650 °C, the experimental steel precipitates (V, Nb, Ti) C phase on the ferrite matrix, the shape is similar to an ellipse, and the particle size is about 80 nm. Figure 15b shows that the (V, Nb, Ti) C precipitated phase in the experimental steel contains Nb/V/Ti, except for the Fe peak, which is mainly the V peak, and the atomic ratio is V:Nb:Ti = 15.26:6.35: 1.05.

Figure 16 shows the distribution characteristics of the precipitated phase of the test steel at the final cooling temperature of 650 °C. According to the Ashby–Orowan precipitation strengthening formula [27,28]:

(3) $$\sigma_{p}=8995\times \frac{f^{\frac{1}{2}}}{d_{p}}ln\left(2.417d_{p}\right)$$

σ*_p_* is the precipitation strengthening increment (MPa); *f* is the volume fraction of carbides (%); *d_p_* is the average diameter of the precipitate particles (nm). It can be seen from formula (3) that the more precipitated It can be seen from formula (3) that the more precipitated phases and the finer the particles the more obvious the strengthening effect and the greater the strengthening contribution to the steel. Taking into account the inconsistency of precipitation distribution and the inability of statistics for some small precipitations, the precipitation effect can only be analyzed qualitatively, and the amount of precipitation enhancement cannot be quantitatively analyzed. The precipitation of (V, Nb, Ti) C was mainly distributed on the ferrite matrix, and the particle size was between 50–100 nm. The size of (Ti, Nb, V) C precipitates greater than 75 nm accounted for about 65% precipitates near the boundary. Through the statistical analysis, (V, Nb, Ti) C had a higher volume fraction of fine precipitates in the steel, and the precipitation strengthening of the steel was greater. According to [29,30], Nb nano precipitates mainly formed during rolling and cooling at austenite grain boundaries or ferrite matrix.

According to the atomic ratio of the above precipitates, the precipitation and growth of carbides or nitrides in the experimental steel containing Nb-V-Ti required three atoms to diffuse into carbides or nitrides. During five passes of hot rolling, the precipitated phases of Nb/V/Ti precipitate in austenite grain boundaries and grains, inhibiting the recrystallization and non-recrystallization of austenite, refining austenite grains, and increasing grain boundaries. In the process of γ → α transformation, the densities of dislocations and defects on the ferrite matrix and grain boundary were high. The precipitates rich in Nb, Ti, and V nucleate and grow on these dislocations and defects rather than on the austenite grain boundary and intragranular nucleation. According to [31,32,33], Nb precipitates may mainly form from strain-induced precipitates in deformed austenite, and then transform into ferrite without recrystallization. No preferred orientation relationship was observed between the transformed ferrite and precipitates, because the precipitates form on the deformed austenite dislocation.

## 4. Discussion

The experimental results (Figure 3 and Figure 7) show that at different final cooling temperatures, the main microstructures in the center of the experimental steel was ferrite and pearlite. The average grain size of ferrite was refined, and the pearlite lamellae were fine and evenly distributed on the ferrite matrix. The main reason was the effect of microalloying element Nb/V/Ti and the hot rolling process. The microalloying element Nb/V/Ti had the following main functions: Ti is combined easily with N to form TiN during hot rolling at a high-temperature zone, which could inhibit austenite recrystallization and grain growth; Nb formed Nb carbide and nitride in high-temperature austenite hot rolling zone and inhibited austenite recrystallization, pinning austenite grain boundary and refining austenite grain size; in the process of γ → α transformation, the transformation of fine grain ferrite provides powerful conditions; and V mainly forms nitrides and carbides in austenite and ferrite region, and VC precipitates at the austenite grain boundary, which provides heterogeneous nucleation point for ferrite, and refined ferrite grains during ferrite transformation.

In hot-rolled microalloyed steels, interstitial elements, such as carbon and nitrogen, plaed an important role in the strengthening of the material matrix of microalloyed steel by forming carbides or carbonitrides [32]. The results of Figure 15 and Figure 16 show that the main precipitates in the experimental steel were (Ti, Nb, V) C, and (V, Nb, Ti) C, which were mainly distributed in the grain boundaries and grains of ferrite. This distribution was mainly attributed to two factors: hot deformation and final cooling temperature. Previous research results [34,35,36] showed that the interfacial precipitation of carbide particles usually occured at higher isothermal holding temperatures, resulting in a significant hardening effect. A large amount of austenite can be generated by the heating deformation of the experimental steel, which increased dislocation and defects. When the steel was cooled slowly at different final cooling temperatures, fine-grain ferrite, and lamellar pearlite were obtained through γ → α transformation. Given the γ → α transformation was a diffusion-controlled process, the growth rate of some ferrite grains formed in the later phase transformation was relatively low and was, thus, suitable for the interface precipitation accompanied by carbides [37].

According to the classical theory of phase transition and compared with homogeneous nucleation, heterogeneous nucleation needed to overcome lower energy. Therefore, it was very difficult for the precipitates of Nb/V/Ti to nucleate uniformly in the experimental steel. A large number of crystal defects and dislocations were produced during austenite hot rolling, which provided a powerful heterogeneous nucleation point for the heterogeneous precipitation of carbides and nitrides and ferrite transformation nucleation of Nb/V/Ti.

## 5. Conclusions

(1) The microstructure of the experimental steel was mainly composed of polygonal ferrite and pearlite. Pearlite was mainly composed of flakes, accompanied by a small amount of short rod-shaped or granular pseudo-pearlite, evenly distributed on the ferrite matrix. The grain size of ferrite and the interlayer spacing of pearlite decreased with the decreased of the final cooling temperature. When the final cooling temperature were 650 °C and 750 °C, the average ferrite grain size were 7.68 μm and 17.07 μm, respectively. The grain sizes were 11 and 9, respectively.
(2) In the experimental steel, the polygonal ferrite grain boundaries had high dislocation orientation, but there were some lower dislocation grain boundaries and subgrain boundaries in the ferrite grains. The high-density dislocations were distributed on the ferrite grain boundaries, which could effectively block the initiation and propagation of cracks.
(3) The main precipitates in the test steel were (Ti, Nb, V) C and (V, Nb, Ti) C. The precipitates were round or narrow, about 70–100 nm in size, and distributed in ferrite grain boundaries and crystals. (V, Nb, Ti)C had obvious precipitation strengthening effect on steel.
(4) With the decrease of the final cooling temperature, the tensile strength and hardness gradually increased. When the final cooling temperature is 750 °C, the fracture types were equiaxed dimples and a few shear planes.

## Figures and Tables

**Table 1:** Chemical composition of experimental steel (wt%).

No	C	Si	Mn	P	S	O	Nb	V	Ti	N
1	0.20	0.38	1.40	0.019	0.012	0.005	0.023	0.088	0.012	0.0083

**Table 2:** Statistics of ferrite grain size and grain size grades of experimental steel.

Temperature/°C	Ferrite Grain Size/μm	Grain Size Grade
650	7.68 ± 1.3	11 ± 0.25
700	10.67 ± 1.5	10 ± 0.25
750	17.07 ± 1.6	9 ± 0.25

## Data Availability

The raw data required to reproduce these findings cannot be shared at this time as the data also form part of an ongoing study.
